# PCSK9 induces a pro-inflammatory response in macrophages

**DOI:** 10.1038/s41598-018-20425-x

**Published:** 2018-02-02

**Authors:** Chiara Ricci, Massimiliano Ruscica, Marina Camera, Laura Rossetti, Chiara Macchi, Alessandra Colciago, Ilaria Zanotti, Maria Giovanna Lupo, Maria Pia Adorni, Arrigo F. G. Cicero, Federica Fogacci, Alberto Corsini, Nicola Ferri

**Affiliations:** 1 Dipartimento di Scienze del Farmaco, Università degli Studi di Padova, Padua, Italy; 2 Dipartimento di Scienze Farmacologiche e Biomolecolari, Università degli Studi di Milano, Milan, Italy; 3 Centro Cardiologico Monzino, Milan, Italy; 4 Dipartimento di Scienze degli Alimenti e del Farmaco, Università di Parma, Parma, Italy; 5 Dipartimento di Scienze Mediche e Chirurgiche, Università di Bologna, Bologna, Italy; 6 Multimedica IRCCS, Milan, Italy

## Abstract

Intraplaque release of inflammatory cytokines from macrophages is implicated in atherogenesis by inducing the proliferation and migration of media smooth muscle cells (SMCs). PCSK9 is present and released by SMCs within the atherosclerotic plaque but its function is still unknown. In the present study, we tested the hypothesis that PCSK9 could elicit a pro-inflammatory effect on macrophages. THP-1-derived macrophages and human primary macrophages were exposed to different concentrations (0.250 ÷ 2.5 µg/ml) of human recombinant PCSK9 (hPCSK9). After 24 h incubation with 2.5 µg/ml PCSK9, a significant induction of IL-1β, IL-6, TNF-α, CXCL2, and MCP1 mRNA, were observed in both cell types. Co-culture of THP-1 macrophages with HepG2 overexpressing hPCSK9 also showed the induction of TNF-α (2.4 ± 0.5 fold) and IL-1β (8.6 ± 1.8 fold) mRNA in macrophages. The effect of hPCSK9 on TNF-α mRNA in murine LDLR^−/−^ bone marrow macrophages (BMM) was significantly impaired as compared to wild-type BMM (4.3 ± 1.6 fold vs 31.1 ± 6.1 fold for LDLR^−/−^ and LDLR^+/+^, respectively). Finally, a positive correlation between PCSK9 and TNF-α plasma levels of healthy adult subjects (males 533, females 537) was observed (B = 8.73, 95%CI 7.54 ÷ 9.93, p < 0.001). Taken together, the present study provides evidence of a pro-inflammatory action of PCSK9 on macrophages, mainly dependent by the LDLR.

## Introduction

The constant and slow development of the atherosclerotic plaque is driven by a failure to resolve the inflammatory response in the arterial wall initiated by the retention of apolipoprotein B (apoB)-containing lipoproteins, mainly the low-density lipoproteins (LDL)^1^. LDL retained in the arterial wall undergo chemical oxidation and then capture, through the scavenger receptors, by infiltrated macrophages, which ultimately become foam cells. The continuing accumulation of LDL fuels not only the formation of foam cells, but also a chronic amplification of the inflammatory response, the main cause of plaque rupture and vascular thrombosis^2^.

The study of the central role of macrophages in atherogenesis led to the observation of the presence of two different macrophage populations that can be grossly divided into pro-inflammatory (M1 cells) and anti-inflammatory (M2 cells) based mainly on *in vitro* criteria^3^. Polarization toward the M1 state is induced by several stimuli *in vitro*, including Toll-like receptor (TLR) ligands (such as lipopolysaccharide, LPS) and interferon γ.

M1 macrophages express several pro-inflammatory mediators, such as inducible nitric oxide synthase, tumor necrosis factor-α (TNF-α), interleukin-1β (IL-1β), IL-6, IL-12, and proteolytic enzymes^3^. The presence of M1 macrophages has been demonstrated in both human and mouse atherosclerotic plaques and is considered the main intravascular source of pro-inflammatory cytokines, responsible for the maintenance of local inflammation and for the extracellular matrix components degradation, thus promoting the disease progression^3^.

Since LDL-cholesterol represents the most validated risk factor for atherosclerosis, several studies have tried to address the relationship between cholesterol and inflammation. Among the different mechanisms that have been postulated, one points toward the increase of plasma membrane cholesterol through the involvement of the TLRs^4,5^. Despite this evidence, a number of studies show opposite results, such as an anti-inflammatory phenotype induced by intracellular cholesterol^6^.

Proprotein convertase subtilisin kexin type 9 (PCSK9) represents the most effective mediator of the LDL receptor degradation and thus a main regulator of the LDL-cholesterol homeostasis^7^. We have previously shown that PCSK9 is present in the atherosclerotic plaque and that the LDL receptor (LDLR) expression in macrophages is influenced by PCSK9 derived from smooth muscle cells^8^.

Interestingly, the PCSK9 knock-down in macrophages partially blunted the induction of pro-inflammatory cytokines in response to oxLDL^9^. The role of PCSK9 as a pro-inflammatory mediator is also supported by the observation that increased LDLR expression is associated with protection from severe sepsis^10^ and, more importantly, the PCSK9 null mice displayed blunted systemic responses to LPS treatment^11^. Furthermore, exogenous overexpression of PCSK9 in macrophages enhanced the response to LPS, increasing the expression of IL1β and TNF-α and decreasing the anti-inflammatory markers Arg1 and IL-10^12^. Finally, the suppression of PCSK9 expression in the atherosclerotic plaques of apoE null mice, by means of lentivirus-mediated PCSK9 shRNA, reduced macrophage infiltration and decreased expression of TNF-α, IL-1β, MCP-1, toll-like receptor 4 (TLR-4) and nuclear factor kappa B (NF-κB)^13^.

In the present study, we investigated the role of PCSK9 on the inflammatory state of macrophages. We demonstrated that recombinant human PCSK9 induced a pro-inflammatory phenotype, by increasing the expression of inflammatory cytokines (TNF-α, IL-1β, IL-6) and chemokines (Monocyte Chemotactic Protein 1, MCP1 and Chemokine C-X-C motif ligand 2, CXCL2) in human THP1 and primary macrophages, as well as in bone marrow derived macrophages (BMM). Similar results were observed by using a co-cultured system with HepG2 cells overexpressing PCSK9 and THP-1-derived macrophages. Finally, by using BMM from genetically modified mice, we demonstrated that the response to PCSK9 is mainly, but not entirely, dependent on the presence of the LDLR. The clinical relevance of our observation is supported by the positive correlation found in the plasma levels of PCSK9 and TNF-alpha of subjects enrolled in the Brisighella Heart Study.

## Material and Methods

### Reagents and antibodies

RPMI media was obtained by Sigma, penicillin, streptomycin, nonessential amino acid solution, Fetal Calf Serum (FCS), disposable culture flasks and petri dishes were from Euroclone. Molecular weight protein standards, SDS, TEMED, ammonium persulfate, glycine, and acrylamide solution (30%T, 2.6%C) were from BIO-RAD Laboratories. BCA assay for determination of protein concentrations was purchased from Thermo Scientific. Tumor Necrosis Factor-α (TNF-α) was purchased from Sigma. Recombinant hPCSK9 was obtained by Vinci Biochem (BPS Biosciences, San Diego CA USA). The recombinant PCSK9 was formulated in 40 mM Tris-HCl, pH 8.0, 110 mM NaCl, 2.2 mM KCl, and 20% glycerol. The endotoxin level of hPCSK9 was 84.8 EU/mL or 47 EU/mg, below the threshold of 0.1 EU/µg requested for cell based assays. The JAK inhibitor JAK1 was purchased from Millipore and fatostatin hydrobromide from Sigma. The JAK inhibitor is a highly potent, ATP-competitive inhibitor of JAK with IC_50_ values equal to 15, 1, 5 and 1 nM for JAK1, 2, 3 and Tyk2, respectively.

### Cell culture

#### THP1-derived macrophages

THP-1 monocyte cells were cultured in RPMI media supplemented with penicillin (10,000 U/mL), streptomycin (10 mg/mL), nonessential amino acid, 10% (FCS and β-mercaptoethanol 0.05 mM. To obtain THP-1 macrophages, THP-1 monocytes were seeded in appropriate multiwell plate and treated with Phorbol 12-myristate 13-acetate (PMA; 3.2 × 10^–7^ M) for 72 h. Then, macrophages were treated for 24 h with PCSK9 0.25, 0.5, 1 and 2.5 ug/ml, using TNF-α 10 ng/ml as positive control.

#### Preparation of Human Macrophages

Human monocytes were obtained from healthy donors who gave their informed consent to participate in the study. Blood sampling - Venous blood samples were obtained by venipuncture of the antecubital vein with a 19 G needle without venous stasis. After discarding the first 4 ml, blood was drawn into 4 ACD-containing vacutainers (Becton Dickinson, CA, USA) for monocyte isolation and into one Z-vacutainer (Becton Dickinson, Ca, USA) for serum preparation. Haemocrome was assessed by Sysmex XS-1000i haematologic analyzer (Kobe).

#### Serum preparation

Serum was obtained by incubating blood at 37 °C for 1 hour followed by centrifugation at 1700 g, 10 minutes, 4 °C and transferred under sterile conditions into a new tube and stored at 4 °C until use.

#### Monocytes isolation

Immediately after withdrawal, ACD-anticoagulated blood was centrifuged at 100 g, 10 minutes at room temperature. The platelet rich-plasma was completely removed and the remaining blood was mixed with RPMI (Lonza, 1640) containing 0,1% L-glutammine (Thermo Fisher), 0.5% Pen-strep (Thermo Fisher), 20% FCS and 0.38% sodium citrate (Sigma) to restore the initial volume of blood in the tubes. Mononuclear cells were isolated by Ficoll-Paque Plus (GE Healthcare, vWR Int.) density centrifugation at 600 g, 20 minutes at room temperature with no brake. Cells were washed with PBS containing 0, 1% glucose (Sigma), 0, 5% BSA (Sigma) and 5 mM EDTA and centrifuged at 830 g for 10 minutes at 4 °C with brake. Cells were further washed twice in PBS without EDTA and centrifuged at 350 g for 10 minutes at 4 °C with brake. Mononuclear cells resuspended in RPMI supplemented with 10% autologous serum, 0, 1% L-glutammine and 0.5% Pen-strep were then plated (100.000 monocytes/100 µl) in a 48 Well Cell Culture Plate and incubated at 37 °C. After 2 hours cells were washed 3 times with PBS in order to remove non-adherent lymphocytes; adherent monocytes were then cultured in the same medium over 7 days at 37 °C (5% CO_2_). Medium was not replaced throughout the culture period.

#### Bone marrow derived macrophages (BMM)

Bone marrow macrophages were collected from the femur and tibia of C57BL/6 or LDLR^−/−^ mice (from Envigo and The Jackson Laboratories respectively). All experiments were conducted in conformity with the Public Health Service Policy on the Humane Care and Use of Laboratory Animals and performed with the approval of the Ethical Committee for Animal Experiments of the University of Parma. After isolation, cells were seeded in 24-well plates in high glucose DMEM containing 30% L-929 cells conditioned medium and 10% FCS and maintained at 37 °C, 5% CO_2_. After 4 days, non-adherent cells were removed and fresh medium was added. After 3 further days, cells were differentiated and treated according to the protocol.

#### HepG2 and THP-1 co-culture

HepG2 or HepG2^PCSK9^ were cultured with THP-1 monocytes by using a transmembrane system (Transwell) of polycarbonate membrane with 0.4 μm pores. HepG2 and HepG2^PCSK9^ cells were seeded in 6-well plates at a density of 6 × 10^5^ cells/well in MEM + 10% FCS. After 3 days, HepG2 and HepG2^PCSK9^ medium was discarded and THP-1 cells, suspended in fresh medium, were added in the transmembrane system to each well seeded with HepG2 or HepG2^PCSK9^, at a density of 2 × 10^5^ cells/well.

#### Quantitative real time PCR (qRT-PCR) assay

Total mRNA was extracted by using iScript Sample Preparation Reagent (BIO-RAD laboratories), according to manufacturer’s instructions. Reverse transcription-polymerase first-strand cDNA synthesis was performed by using the iScript cDNA synthesis Kit (BIO-RAD laboratories) and for qRT-PCR were used the Kit Thermo Sybr Green/ROX qPCR Master Mix (Carlo Erba Reagents S.r.l.) and specific primers of the genes of interest (described below). The analyses were performed with the ABI Prism® 7000 Sequence Detection System (Applied Biosystems), as previously described^14^. PCR cycling conditions were as follows: 94 °C for 3 min, 40 cycles at 94 °C for 15 s, and 60 °C for 1 min. Data were expressed as Ct values and used for the relative quantification of targets with the ΔΔCt calculation. Primer sequences used for qRT-PCR analysis are shown in Table 1.

**Table 1:** Primer sequence utilized for the qRT-PCR analysis.

**Primer**	**Forward**	**Reverse**
18S	5′-CGGCTACCACATCCACGGAA-3′	5′-CCTGTATTGTTATTTTTCGTCACTACC-3′
***human***		
hIL-6	5′-GGTACATCCTCGACGGCATCT-3′	5′-GTGCCTCTTTGCTGCTTTCAC
hIL-1β	5′-ATGCACCTGTACGATCACTG-3′	5′-ACAAAGGACATGGAGAACACC-3′
hTNF-α	5′-ACTTTGGAGTGATCGGCC-3′	5′-GCTTGAGGGTTTGCTACAAC-3′
hCXCL2	5′-CGCCCATGGTTAAGAAAATCA-3′	5′-CCTTCTGGTCAGTTGGATTTGC-3′
hMCP1	5′-CGCCTCCAGCATGAAAGTCT-3′	5′-GGAATGAAGGTGGCTGCTATG-3′
hPCSK9	5′-CCTGCGCGTGCTCAACT-3′	5′-GCTGGCTTTTCCGAAACTCT-3′
hLDLR	5′-GTGTCACAGCGGCG-3′	5′-CGCACTCTTTGATG-3′
***mouse***		
mIL-6	5′-GAGGATACCACTCCCAACAGACC-3′	5′-AAGTGCATCATCGTTGTTCATACA-3′
mIL-1β	5′-CAACCAACAAGTGATATTCTCCATG-3′	5′-GATCCACACTCTCCAGCTGCA-3′
mTNF-α	5′-CCCTCACACTCAGATCATCTTCT-3′	5′-GCTACGACGTGGGCTACAG-3′
mCXCL2	5′-CCAAGGGTTGACTTCAAGAAC-3′	5′-AGCGAGGCACATCAGGTACG-3′
mMCP-1	5′-ACCACAGTCCATGCCATCAC-3′	5′-TTGAGGTGGTTGTGGAAAAG-3′
mLDLR	5′-GTGTGACCGTGAACATGACTGC-3′	5′-CACTCCCCACTGTGACACTTGA-3′

#### ELISA assay

Conditioned media from treated cells were collected and used for the protein quantification of PCSK9, IL-6 and TNF-α by ELISA kit (Quantikine ELISA Kit, R&D Systems), as previously described^15^.

#### Western Blot analysis

Total cytosolic protein extracts were obtained by collecting cells in 200 μl of Mammalian Protein Extraction Reagents (Thermo Fisher Scientific) containing a cocktail of protease and phosphatase inhibitors (Roche Diagnostics S.p.A.). Molecular mass marker (Novex® Sharp Protein Standard, Invitrogen^TM^; Life Technologies Europe BV) and proteins were separated through 10–12% SDS-PAGE gel. Proteins were then transferred to a nitrocellulose membrane and blocked with albumin buffer containing 0.05% of Tween20. Incubation with primary antibodies occurred overnight at 4 °C with the following antibodies: LDLR rabbit Polyclonal Antibody 1:200 (Cayman Chemical) and α-tubulin 1:5000 (Sigma). Membrane was incubated with anti-mouse and anti-rabbit peroxidase-conjugated secondary antibodies (1:5000) (Jackson ImmunoResearch Lab). Immunoreactive bands were detected by acquiring images with Odyssey® (LI-COR Biosciences). Densitometric readings were evaluated using the Image Studio Software, as previously described^16^.

#### Immunocytochemistry

Cells were fixed in 4% paraformaldehyde at room temperature for 10 min, permeabilized in 0.1% Triton X-100 in PBS for 5 min, and incubated for 1 h with 1% bovine serum albumin (BSA). Cells were then incubated with primary antibody anti NF-κB p65 (Rabbit polyclonal GeneTex) for 1 h at room temperature, followed by three washes with PBS and subsequent incubation with Alexafluor®−568 anti-rabbit antibody. Cells were then washed four times with PBS, incubated with DAPI solution for 5 min, mount and coversliped with Vectashield. Immunostaining of cells was analyzed by fluorescent confocal microscope (Zeiss LSM 800).

#### Subjects

Study participants were patients enrolled in the Brisighella Heart Study. All the involved subjects have signed an informed consent form. The Brisighella Heart Study protocol and its substudies have been evaluated and approved by the Ethical Board of the S. Orsola-Malpighi University Hospital (Bologna, Italy). All experiments were performed in accordance with relevant guidelines and regulations. We selected a sample of overall healthy adult subjects (M: 533, F: 537), after exclusion of active smokers, subjects affected by chronic inflammatory disorders (including atopic diseases) and subjects chronically assuming non-steroidal antinflammatory drugs, systemic corticosteroids, or immunosuppressants^17^. A Pearson’s bivariate correlation followed by a multiple linear regression have been carried out to evaluate the potential relationship between TNF-α and PCSK9 plasma levels in humans.

### Statistical analysis

Statistical analysis was performed using the Prism statistical analysis package version 5.01 (GraphPad Software). Data are given as mean ± SD of three independent experiments. When possible, p-values were determined by Student’s t-test. Otherwise, differences between treatment groups were evaluated by 1-way ANOVA. A probability value of p < 0.05 was considered statistically significant.

## Results

**Figure 1:**
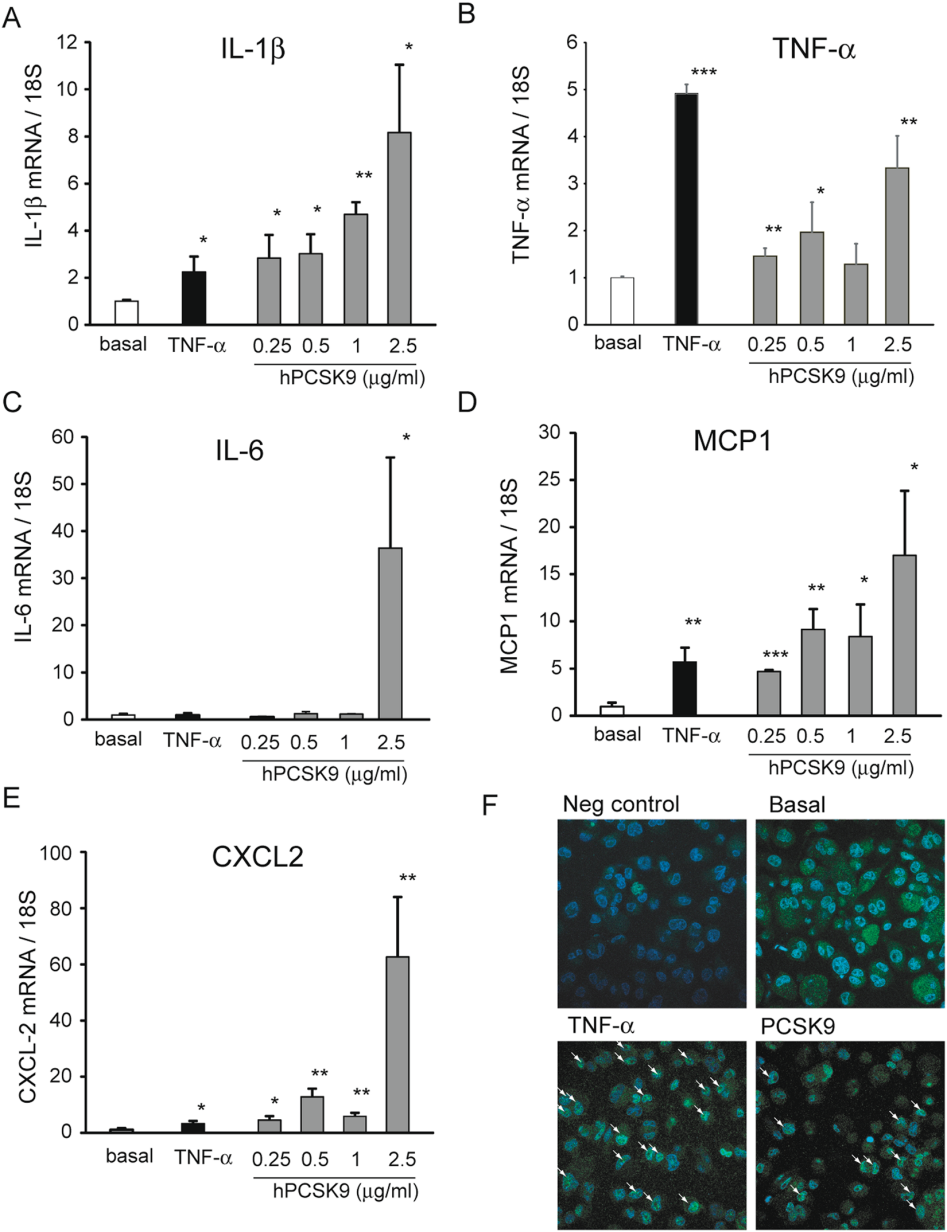
Recombinant hPCSK9 induces the expression of pro-inflammatory cytokines and chemokines in THP-1 derived macrophages. THP-1 macrophages were incubated for 24 h with TNF-α 10 ng/ml (as positive control) or different concentrations of recombinant hPCSK9 (0.25, 0.5, 1 and 2.5 μg/ml). At the end of the incubation, total RNA was extracted and (**A**) IL-1β, (**B**) TNF-α, (**C**) IL-6, (**D**) MCP-1 and (**E**) CXCL2 mRNA expression was determined by qRT-PCR. (**F**) THP-1 macrophages were incubated with TNF-α 10 ng/ml (as positive control) and recombinant hPCSK9 (2.5 μg/ml). After 24 h cells were fixed and immunostaining was performed for NF-κB p65 (blue: nuclei; green: p65 NF-κB). Arrows indicate cells with positive nuclear staining of p65. Data are given as mean ± SD of three independent experiments. Differences vs basal were assessed by Student’s t-test: *p < 0.05; **p < 0.01; ***p < 0.001.

To test the hypothesis that PCSK9 could have a pro-inflammatory effect on macrophages, we first performed a series of experiments with macrophages derived from human monocyte cell line THP-1 incubated with increasing concentrations of human recombinant PCSK9. The incubation for 24 h induced the mRNA levels of pro-inflammatory cytokines IL-1β, IL-6 and TNF-α, markers of M1 phenotype (Fig. 1A–C). In particular, the effect of hPCSK9 on IL-1β appears to be concentration-dependent, with a significant induction starting from 250 ng/ml (Fig. 1A). The mRNA levels of TNF-α also increased in a concentration-dependent manner, although we observed a consistent lower effect at 1 µg/ml than lower concentrations and a maximal effect at 2.5 µg/ml (3.3 ± 0.7 fold) (Fig. 1B). Differently, hPCSK9 strongly induced IL-6 mRNA levels exclusively at 2.5 µg/ml (36.4 ± 19.3 fold), with no effect at 250 ng/ml, 0.5 µg/ml and 1 µg/ml (Fig. 1C).

The determination of mRNA levels of monocyte chemokines, such as MCP-1 and CXCL2, also revealed a positive effect of hPCSK9 (Fig. 1D,E), with a concentration dependent response for MCP-1 (similar to IL1-β) and a strong induction at 2.5 µg/ml for CXCL2 (similar to TNF-α). In all the experiments, THP1 macrophages were also incubated with TNF-α (10 ng/ml) as positive control. Interestingly, the effect of 250 ng/ml hPCSK9, thus within its physiological plasma concentrations (median 270 ng/mL, ranging from 91 to 804 ng/ml^18^), exerted a similar pro-inflammatory effect than TNF-α.

Since transcription of the majority of proinflammatory cytokines are under the control of NF-κB transcription factor, we investigated the effect of PCSK9 on the nuclear localization of p65 subunit in THP-1 derived macrophages. As expected, the incubation with TNF-α induced a marked response of NF-κB activation with positive nuclear staining of p65 cells NF-κB in approximately 30% of the cells, while no nuclear staining was observed under basal condition. Interestingly, the incubation with 2.5 µg/ml of PCSK9 induced a significant NF-κB activation, with 16% of the cells with positive nuclear staining for p65 (Fig. 1F).

**Figure 2:**
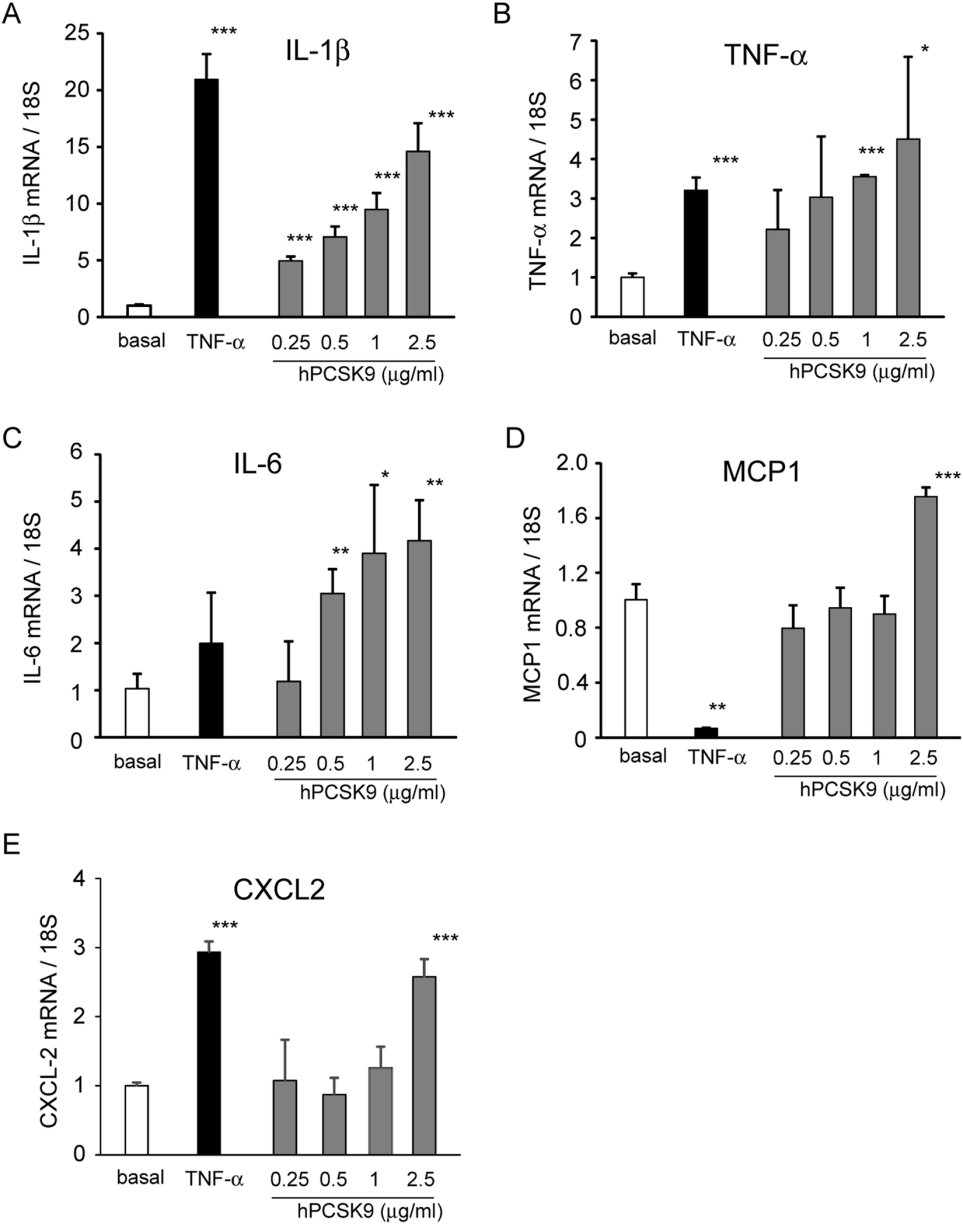
Recombinant hPCSK9 induces the expression of pro-inflammatory cytokines and chemokines in human macrophages. Human macrophages were incubated for 24 h with TNF-α 10ng/ml (as positive control) or different concentrations of recombinant hPCSK9 (0.25, 0.5, 1 and 2.5 μg/ml). At the end of the incubation, total RNA was extracted and (**A**) IL-1β, (**B**) IL-6, (**C**) TNF-α, (**D**) MCP-1 and (**E**) CXCL2 mRNA expression was determined by qRT-PCR. Data are given as mean ± SD of three independent experiments. Differences vs basal were assessed by Student’s t-test: *p < 0.05; **p < 0.01; ***p < 0.001.

To extend our observation, we performed the same analysis on human macrophages derived from blood of healthy volunteers. A concentration-dependent effect of hPCSK9 on mRNA levels of IL-1β, IL-6 and TNF-α was observed, with a significant induction at 1 µg/ml and 2.5 µg/ml for all genes (Fig. 2). As observed with THP-1, hPCSK9 induced IL-1β also at 250 ng/ml. A significant increase of MCP1 and CXCL2 mRNA levels were observed after incubation with 2.5 µg/ml of hPCSK9 (Fig. 2).

**Figure 3:**
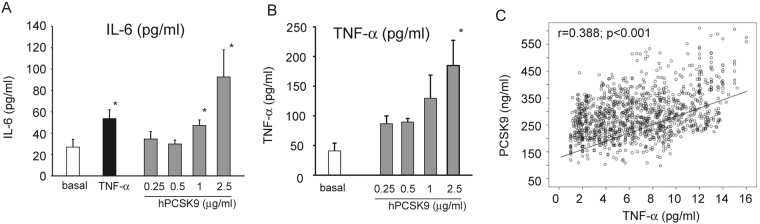
Effect of recombinant hPCSK9 on the release of IL-6 and TNF-α from human macrophages. Human monocytes were seeded in 6 well plates in RPMI containing 10% of the volunteer autologous serum and incubated for 1 week. Human macrophages were then incubated for 24 h with TNF-α 10 ng/ml (as positive control) or different concentrations of recombinant hPCSK9 (0.25, 0.5, 1 and 2.5 μg/ml). IL-6 (**A**) and TNF-α (**B**) protein amount was then assessed by ELISA assay on collected media. Results were expressed as pg of protein for ml of media. (**C**) Relationship between TNF-α and PCSK9 serum levels determined by ELISA assay from human samples. Data of panel A and B are given as mean ± SD of three independent experiments. Differences vs basal were assessed by Student’s t-test: *p < 0.05; **p < 0.01; ***p < 0.001.

To further validate our observation, we determined the effect of hPCSK9 on IL1-β and IL-6 released by human macrophage in the cultured media, by ELISA assay. As shown in Fig. 3, 24 h incubation with 2.5 µg/ml hPCSK9 significantly increased both IL-6 and TNF-α concentration (3.6 ± 1.3 fold and 4.5 ± 0.2 fold, respectively).

Overall, recombinant hPCSK9 induced a pro-inflammatory response in both THP1 and human macrophages, showing a robust induction of mRNA of IL-1β, IL-6, TNF-α, MCP1, CXCL2, and an increased IL-1β and TNF-α released from human macrophages.

To further corroborate our results, we investigated a possible correlation between PCSK9 and TNF-α plasma levels from a selected overall healthy adult subjects (males 533, females 537) enrolled in the Brisighella Heart Study^17^. The main characteristics of the selected subjects are summarized in Table 2. The Pearson’s bivariate correlation between TNF-α and PCSK9 showed a correlation coefficient of 0.388 with a p < 0.001 (Fig. 3C). After adjustment for age, gender, and BMI, in the multiple linear analysis, TNF-α and PCSK9 plasma levels were significantly related (B = 8.73, 95% CI 7.54 ÷ 9.93, p < 0.001) (Fig. 3C). This evidence further supports a relationship between chronic inflammation and PCSK9 levels.

**Table 2:** Main Characteristics of the Selected Participants.

	Mean ± SD
AGE	57.66 ± 11.77
BMI	23.33 ± 3.46
SBP	130.02 ± 11.37
DBP	67.78 ± 7.12
TC	218.12 ± 19.38
TG	118.29 ± 69.35
HDL-C	51.97 ± 5.54
LDL-C	141.58 ± 18.31
FPG	93.24 ± 5.61
SUA	5.215 ± 1.29
GOT	23.17 ± 7.59
GPT	24.13 ± 9.32
gGT	25.67 ± 13.47
Creatinine	1.03 ± 0.19
eGFR (CKD-EPI)	81.30 ± 15.41
PCSK9 (ng/ml)	286.25 ± 81.14
TNF alpha (pg/ml)	6.69 ± 3.60

**Figure 4:**
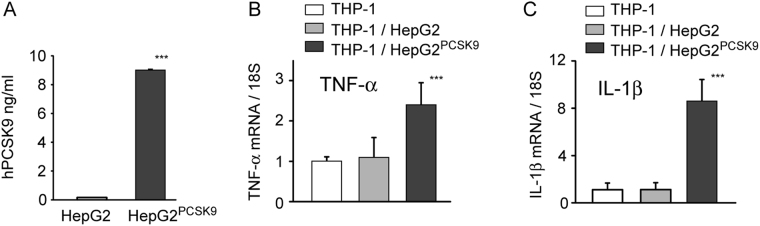
PCSK9 released from HepG2 increased TNF-α and IL-1β gene expression in THP-1 derived macrophages. THP-1 macrophages were co-cultured with HepG2 or HepG2^PCSK9^ seeded on top of the transwell system. After 24 h, total RNA was extracted from THP-1 macrophages and TNF-α and IL-1β gene expression was evaluated by qRT-PCR. Data are given as mean ± SD of three independent experiments. Differences were assessed by Student’s t-test: *p < 0.05; **p < 0.01; ***p < 0.001.

To further establish the pro-inflammatory effect of PCSK9, we performed co-cultured experiments of THP-1 macrophages and HepG2 control or overexpressing PCSK9. HepG2 were transduced with pBM-IRES-PURO retrovirus encoding control vector (PURO) or human PCSK9. After puromycin selection, we measured the amount of PCSK9 released in the cultured media by ELISA assay. While control HepG2 cells released 1.7 ng/ml of PCSK9, the HepG2^PCSK9^ reached approximately 10 ng/ml (9.0 ± 1.0 ng/ml) (Fig. 4A). The co-culture with THP1 macrophage revealed that the exposure to conditioned media from HepG2^PCSK9^ significantly induced the pro-inflammatory genes TNF-α and IL-1β by 2.4 ± 0.5 fold and 8.6 ± 1.8 fold, respectively, as compared to HepG2 (Fig. 4B,C).

**Figure 5:**
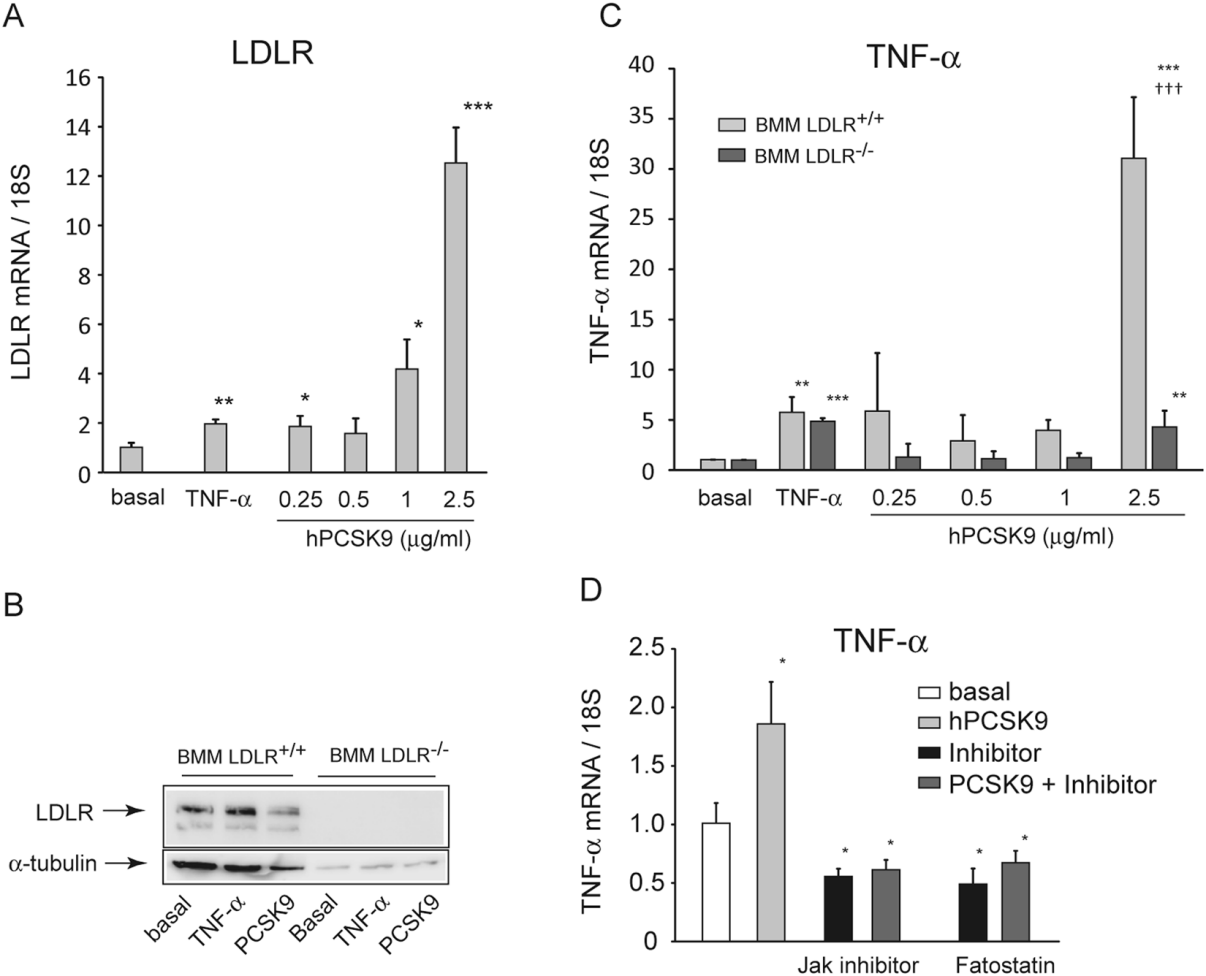
Effect of recombinant hPCSK9 on TNF-α mRNA expression in LDLR^−/−^ BMM. LDLR^+/+^ and LDLR^−/−^ BMM were incubated with TNF-α 10 ng/ml (as positive control) or different concentrations of recombinant hPCSK9 (0.25, 0.5, 1 and 2.5 μg/ml). After 24 h, total RNA and protein extracts were prepared. LDLR receptor mRNA (**A**) and protein (**B**) expressions were evaluated by qRT-PCR and western blotting, respectively. (**C**) TNF-α mRNA levels were determined by qRT-PCR. (**D**) THP-1 macrophages were incubated with hPCSK9 (2.5 μg/ml) in the presence or absence of the JAK inhibitor (10 µM) and fatostatin (100 µM). Data are given as mean ± SD of three independent experiments. Differences vs basal (*) and between genotypes (†) were assessed by 1-way ANOVA: *p < 0.05; **p < 0.01; ***p < 0.001. ^†^p < 0.05; ^††^p < 0.01; ^†††^p < 0.001.

Since the main molecular target of PCSK9 is the LDLR, we performed the same analysis on bone marrow macrophages (BMM) isolated from C57BL/6 and LDLR^−/−^ mice. The incubation of BMM LDLR^+/+^ with increasing concentrations of hPCSK9 determined a significant induction of the LDLR mRNA (Fig. 5A), and a downregulation of LDLR (−33% at 2.5 µg/ml of hPCSK9), as assessed by western blot analysis (Fig. 5B). Under the same experimental conditions, BMM LDLR^+/+^ respond to 2.5 µg/ml hPCSK9 by increasing the expression of TNF-α (31.1 ± 6.1 fold), while no significant effect was observed at lowest PCSK9 concentrations (Fig. 5C). Interestingly, the BMM LDLR^−/−^ showed only a marginal increase of TNF-α expression (4.3 ± 1.6 fold), which is significantly lower than that observed in BMM LDLR^+/+^. The different response to hPCSK9 is further validated by the fact that both macrophage types respond at similar extent to TNF-α (5.7 ± 1.5 vs 4.9 ± 0.3 fold in BMM LDLR^+/+^ and BMM LDLR^−/−^, respectively) (Fig. 5C). Taken together, the present results suggest that the pro-inflammatory response of PCSK9 on macrophages is mainly, but not exclusively, dependent on the presence of the LDLR. Interestingly, the pharmacological inhibition of Janus Kinase (JAK)/Signal Transducer and Activator of Transcription (STAT) signaling, by a JAK inhibitor, and the SREBP pathway, by fatostatin, completely prevented the induction of the TNF-α mRNA by PCSK9 (Fig. 5D).

## Discussion

In the present report, we demonstrated that human recombinant PCSK9 drives an inflammatory response on macrophages by inducing the pro-inflammatory cytokines TNF-α, IL-1 and IL-6, and the chemokines MCP-1 and CXCL2. This effect was observed in THP-1, human primary, and murine bone marrow-derived macrophages. In addition, the inflammatory response was observed when THP-1 macrophages were co-cultured with HepG2 cells overexpressing PCSK9. This data excludes the possibility that endotoxin contamination of human recombinant PCSK9 can be responsible for the pro-inflammatory effect observed. Finally, we found a positive correlation between plasma levels of PCSK9 and TNF-α, in a population of overall healthy subjects^17,19^, an observation that further supports the role of PCSK9 on inflammation.

Macrophages present in the atherosclerotic plaque are derived, mainly but not exclusively, from circulating monocytes that differentiated into macrophages within the arterial wall^20^. The main function of macrophages is to scavenge lipoprotein particles, and eventually to become foam cells^21^, which then contribute to a local release of inflammatory molecules and factors that further promote lipoprotein retention and extracellular matrix degradation^22^. Macrophages are very heterogeneous cells that can polarized to different phenotypes in response to the environmental cues encountered^23^. Local cytokines, as well as oxidized LDL, have a very potent effect on macrophage polarization^24^ and our data show that PCSK9 is also capable to influence the pro-inflammatory status of macrophages.

The discovery that PCSK9 is present in the atherosclerotic plaque argues a possible local effect on SMCs, macrophages and endothelial cells^8^. PCSK9 can reach the plaque from the bloodstream, as it binds to lipoproteins^25^, or can be synthesized within the arterial wall from SMCs^8,26^. Both sources can be theoretically possible since both protein and mRNA PCSK9 have been detected in the human atherosclerotic plaques^8,25^. Although the intraplaque concentration of PCSK9 is not known, it is important to underlying that we observed a pro-inflammatory effect on macrophages at relatively low concentrations, starting from 250 ng/ml, thus even below the mean plasma levels found in observational studies^19^.

The translation of our findings into experimental atherosclerosis and human pathology still needs to be determined. However, alirocumab, an anti PCSK9 monoclonal antibody, has shown to influence the atherosclerotic plaque development towards a more stable characteristic with reduced macrophage content and higher deposition of extracellular matrix^27^. In addition, the treatment with anti PCSK9 monoclonal antibodies reduces CCR2 expression and the migratory capacity of circulating monocytes in familial hypercholesterolaemia (FH) patients^28^. This effect seems to be due to a reduction of both LDL-C circulating levels and intracellular lipid accumulation. This evidence suggests a possible association between LDL-C lowering and anti-inflammatory effect on circulating monocytes^28^. In addition, the ATHEROREMO-IVUS study demonstrated a linear relationship between PCSK9 plasma levels and both fraction and amount of necrotic core tissue in coronary atherosclerosis, independently of serum LDL-cholesterol levels^18^.

In the attempt to define the molecular mechanism of the pro-inflammatory effect of PCSK9, we have utilized macrophages derived from LDLR null mice. From these analysis, we observed that in presence of the LDLR the induction of TNF-α by PCSK9 is massive. However, a significant effect was still present in BMM LDLR^−/−^. Thus, additional receptors targeted by PCSK9, such as CD36^29^, VLDLR, LRP-1^30^ or ApoER2^31^, could participate in the pro-inflammatory response. Our data are in agreement with *in vitro*^12^ and *in vivo* experimental settings^32^. By using chemical inhibitors, we also demonstrated that the proinflammatory effect of PCSK9 is dependent on the activation of JAK and SREBP pathways. Although the regulation of the intracellular molecular pathways by PCSK9 requires further investigations, it is relevant to note that THP-1 derived macrophages showed a nuclear localization of p65 NF-κB proinflammatory transcription factor, although at lower extent than the TNF-α. This evidence further support a pro-inflammatory function of PCSK9 as potential cytokine.

Native LDL, a natural LDLR ligand, has recently shown to promote the differentiation of monocytes to macrophages^33^. In particular, LDL facilitated the M1 polarization, blocked the ability of monocytes to polarize into M2 macrophages, and enhanced the inflammatory M1 response^33^. From this evidence, it is conceivable to hypothesize that LDL particles and PCSK9 activate similar intracellular signaling pathways by interacting with the LDLR, which induces a pro-inflammatory effect on macrophages.

By using a mouse model of bone marrow transplantation, Fazio *et al*., have investigated the effect of specific PCSK9 overexpression in macrophages on experimental atherosclerosis^12^. Despite no differences in lipid profile, the lesions of mice with macrophages expressing the PCSK9 transgene, showed an LDLR-dependent increase of pro-inflammatory monocytes^12^. Moreover, the expression of PCSK9 increased the presence of CD11b- and Ly6C^hi^-positive cells in spleens of apoE^−/−^ mice and significantly improved the pro-inflammatory response to lipopolysaccharide (LPS)^12^. Our findings are in agreement with this study and further extend the observation of pro-inflammatory properties of PCSK9 in the absence of a co-stimulus, such as LPS. On this matter, it is important to mention that lipoproteins and their receptors play a key role during sepsis as they favor the hepatic clearance of endotoxins, such as LPS. Indeed, PCSK9 null mice show higher hepatic LDLR expression and improved clearance of bacterial endotoxin via the LDLR pathway, and a higher resistance to LPS-induced septic shock^11^.

In our study, together with pro-inflammatory cytokines, PCSK9 significantly induced two chemokines relevant for monocyte recruitment in the atherosclerotic plaque, such as MCP-1^34^ and CXCL2^35^. From this *in vitro* observation, it is tempting to speculate that PCSK9 could promote a local inflammatory response by reinforcing the recruitment of circulating monocytes and neutrophils in the atherosclerotic plaque. Nevertheless, additional and more specific experiments need to be performed in order to demonstrate this action.

In the attempt to translate our findings to a clinical setting, we performed a correlation analysis between the plasma levels of PCSK9 and TNF-α in a population of healthy subjects recruited in the Brisighella Heart Study^17^. For the analysis, we excluded subjects affected by chronic inflammatory disorders, and those chronically assuming non-steroidal antinflammatory drugs, systemic corticosteroids, or immunosuppressants. Interestingly, a positive association between the two factors was found, supporting the possibility that either TNF-α drives the expression of PCSK9^36^, or vice versa PCSK9 induces TNF-α.

In conclusion, in the present study we provided evidence for a direct pro-inflammatory effect of PCSK9 on macrophages. A pathophysiological relevance of this *in vitro* observation requires further investigations.

## References

[1] Williams KJ, Tabas I (1995). The response-to-retention hypothesis of early atherogenesis. Arterioscler Thromb Vasc Biol.

[2] Hansson GK, Libby P, Tabas I (2015). Inflammation and plaque vulnerability. Journal of internal medicine.

[3] Gordon S, Martinez FO (2010). Alternative activation of macrophages: mechanism and functions. Immunity.

[4] Yvan-Charvet L (2008). Increased inflammatory gene expression in ABC transporter-deficient macrophages: free cholesterol accumulation, increased signaling via toll-like receptors, and neutrophil infiltration of atherosclerotic lesions. Circulation.

[5] Zhu X (2008). Increased cellular free cholesterol in macrophage-specific Abca1 knock-out mice enhances pro-inflammatory response of macrophages. J Biol Chem.

[6] Spann NJ (2012). Regulated accumulation of desmosterol integrates macrophage lipid metabolism and inflammatory responses. Cell.

[7] Ferri N, Ruscica M (2016). Proprotein convertase subtilisin/kexin type 9 (PCSK9) and metabolic syndrome: insights on insulin resistance, inflammation, and atherogenic dyslipidemia. Endocrine.

[8] Ferri N (2012). Proprotein convertase subtilisin kexin type 9 (PCSK9) secreted by cultured smooth muscle cells reduces macrophages LDLR levels. Atherosclerosis.

[9] Tang Z (2012). PCSK9 siRNA suppresses the inflammatory response induced by oxLDL through inhibition of NF-kappaB activation in THP-1-derived macrophages. International journal of molecular medicine.

[10] dos Santos C, Marshall JC (2014). Bridging lipid metabolism and innate host defense. Science translational medicine.

[11] Walley KR (2014). PCSK9 is a critical regulator of the innate immune response and septic shock outcome. Science translational medicine.

[12] Giunzioni I (2016). Local effects of human PCSK9 on the atherosclerotic lesion. The Journal of pathology.

[13] Tang ZH (2017). New role of PCSK9 in atherosclerotic inflammation promotion involving the TLR4/NF-kappaB pathway. Atherosclerosis.

[14] Ferri N (2007). Simvastatin reduces MMP1 expression in human smooth muscle cells cultured on polymerized collagen by inhibiting Rac1 activation. Arterioscler Thromb Vasc Biol.

[15] Ruscica M (2016). Liver fat accumulation is associated with circulating PCSK9. Annals of medicine.

[16] Ferri N (2016). PCSK9 knock-out mice are protected from neointimal formation in response to perivascular carotid collar placement. Atherosclerosis.

[17] Cicero AF (2016). NoSAS score associated with arterial stiffness in a large cohort of healthy individuals. The Lancet. Respiratory medicine.

[18] Cheng JM (2016). PCSK9 in relation to coronary plaque inflammation: Results of the ATHEROREMO-IVUS study. Atherosclerosis.

[19] Ruscica, M. *et al*. Circulating Levels of Proprotein Convertase Subtilisin/Kexin Type 9 and Arterial Stiffness in a Large Population Sample: Data From the Brisighella Heart Study. *Journal of the American Heart Association* **6**, 10.1161/JAHA.117.005764 (2017). 10.1161/JAHA.117.005764 PMC5524108 28468788

[20] Jenkins SJ (2011). Local macrophage proliferation, rather than recruitment from the blood, is a signature of TH2 inflammation. Science.

[21] Lusis AJ (2000). Atherosclerosis. Nature.

[22] Libby P (2002). Inflammation in atherosclerosis. Nature.

[23] Porcheray F (2005). Macrophage activation switching: an asset for the resolution of inflammation. Clinical and experimental immunology.

[24] Hirose K (2011). Different responses to oxidized low-density lipoproteins in human polarized macrophages. Lipids in health and disease.

[25] Kosenko T, Golder M, Leblond G, Weng W, Lagace TA (2013). Low density lipoprotein binds to proprotein convertase subtilisin/kexin type-9 (PCSK9) in human plasma and inhibits PCSK9-mediated low density lipoprotein receptor degradation. J Biol Chem.

[26] Perisic L (2013). Profiling of atherosclerotic lesions by gene and tissue microarrays reveals PCSK6 as a novel protease in unstable carotid atherosclerosis. Arterioscler Thromb Vasc Biol.

[27] Van der Hoorn, J. W. A., Kuhnast, S., Pieterman, E. J., Van den Hoek, A. M. & Sasiela, W. J. Alirocumab, monoclonal antibody to PCSK9, dose-dependently decreases atherosclerosis, improves plaque stability and shows additive effects with atorvastatin in apoE*3Leiden.CETP mice. *Atherosclerosis Abstract from EAS Congress held in Madrid* (2014).

[28] Bernelot Moens SJ (2017). PCSK9 monoclonal antibodies reverse the pro-inflammatory profile of monocytes in familial hypercholesterolaemia. Eur Heart J.

[29] Demers A (2015). PCSK9 Induces CD36 Degradation and Affects Long-Chain Fatty Acid Uptake and Triglyceride Metabolism in Adipocytes and in Mouse Liver. Arterioscler Thromb Vasc Biol.

[30] Canuel M (2013). Proprotein convertase subtilisin/kexin type 9 (PCSK9) can mediate degradation of the low density lipoprotein receptor-related protein 1 (LRP-1). PloS one.

[31] Poirier S (2008). The proprotein convertase PCSK9 induces the degradation of low density lipoprotein receptor (LDLR) and its closest family members VLDLR and ApoER2. J Biol Chem.

[32] Denis M (2012). Gene inactivation of proprotein convertase subtilisin/kexin type 9 reduces atherosclerosis in mice. Circulation.

[33] Al-Sharea A (2016). Native LDL promotes differentiation of human monocytes to macrophages with an inflammatory phenotype. Thromb Haemost.

[34] Raines EW, Ferri N (2005). Thematic review series: The immune system and atherogenesis. Cytokines affecting endothelial and smooth muscle cells in vascular disease. J Lipid Res.

[35] Liehn EA (2010). A new monocyte chemotactic protein-1/chemokine CC motif ligand-2 competitor limiting neointima formation and myocardial ischemia/reperfusion injury in mice. J Am Coll Cardiol.

[36] Ruscica M (2016). Suppressor of Cytokine Signaling-3 (SOCS-3) Induces Proprotein Convertase Subtilisin Kexin Type 9 (PCSK9) Expression in Hepatic HepG2 Cell Line. J Biol Chem.

